# Effectiveness of a formulary system on prophylactic oral proton pump inhibitors for drug-induced peptic ulcer in a Japanese tertiary hospital: an interrupted time series analysis

**DOI:** 10.1186/s40780-025-00459-w

**Published:** 2025-06-18

**Authors:** Ryo Ishida, Natsuki Yamada, Kana Sato, Ayako Maekawa, Takehito Kamei, Shinsuke Akagi, Hidenori Tokuda, Hironobu Tokumasu, Motowo Mizuno, Kazunobu Takayanagi

**Affiliations:** 1 Clinical Research Center, Kurashiki Central Hospital, Miwa1-1-1, Kurashiki-city, Okayama 710-8602 Japan; 2 Division of Pharmacy, Kurashiki Central Hospital, Miwa1-1-1, Kurashiki-city, Okayama 710-8602 Japan; 3 Laboratory of Clinical Pharmacy, Faculty of Pharmacy, Shujitsu University, 1-6-1Nishigawara, Naka-ku, Okayama, 703-8516 Japan; 4 Department of Gastroenterology and Hepatology, Kurashiki Central Hospital, Miwa1-1-1, Kurashiki-city, Okayama 710-8602 Japan

**Keywords:** Hospital formulary, Proton pump inhibitor, Drug-induced peptic ulcer, Interrupted time series, Cost-analysis

## Abstract

**Background:**

Pharmacy formulary systems have been established recently in various fields of pharmacotherapies. We evaluated the effectiveness of pharmacy formulary interventions on proton pump inhibitors (PPI) used to prevent drug-induced peptic ulcers in a Japanese tertiary hospital.

**Methods:**

A retrospective cohort study was conducted in Kurashiki Central Hospital. A pharmacy formulary system of PPIs was implemented on October 1, 2020. Between April 2020 and March 2021, six months before and after the implementation date of the formulary system, inpatients aged ≥ 18 years were included if they newly received drugs (low-dose aspirin, anti-platelets, or non-steroidal anti-inflammatory drugs) recommended for prophylactic PPI use for peptic ulcers within seven days from hospital admission. Eligible patients were divided into two groups based on the implementation date, and changes in PPI prescription patterns were evaluated by interrupted time series analysis, along with the risk of drug-induced peptic ulcers and drug costs.

**Results:**

In total, 2,449 inpatients were included. The median age of the pre- and post-formulary group was 60 and 58 years, respectively. The proportion of the targeted PPI prescription increased by 8.75% (95% confidence interval (CI); 0.12, 17.38) in level change, without increased risk of drug-induced peptic ulcers (risk difference -0.41%, 95% CI; -1.38, 0.55). The distribution of medication days in the two groups was similar, and $1,000 per 90 patient days was saved on drug costs.

**Conclusion:**

The formulary system on oral PPIs in a Japanese tertiary hospital contributed to a positive level change in the prescription patterns, without increased risk of drug-induced peptic ulcers. Although slight, the drug costs were saved.

**Supplementary Information:**

The online version contains supplementary material available at 10.1186/s40780-025-00459-w.

## Background

The guidelines published in 2008 and 2021 by the American Society of Health-system Pharmacists delineate the principles of a modern formulary system [1, 2]. Accordingly, formulary systems should establish pharmacotherapeutic policies and identify the most appropriate and cost-effective pharmacotherapy in each medical setting. Some studies have reported the impacts of the formulary systems in various fields of pharmacotherapy [3–6]. Specifically for proton pump inhibitors (PPI), strategies based on the theories of pharmacology, pharmacodynamics, clinical efficacy, safety, and medical economics have been proposed in the field of critical care [7, 8]. Moreover, in other settings, some studies have focused on the change in prescription patterns [9–11]. Although previous studies have confirmed the effectiveness of the formulary system on prescribing patterns and cost changes, the targeted population and outcomes of each study vary due to differences in social backgrounds where the system has been proposed. Thus, each formulary setting merits investigation.

In Japan, guidelines for the use of PPI were published in 2020 for peptic ulcers [12] and in 2015 for gastroesophageal reflux disease [13] (the latter guideline was revised in 2021). Referring to these guidelines and considering the available agents including a generic formulation, we developed a formulary system on oral PPI use in Kurashiki Central Hospital (KCH), a Japanese tertiary hospital with 1,172 beds.

To evaluate the effectiveness of the formulary system, including that on the clinical outcome of PPI use, we investigated changes after intervention using the formulary system in patients receiving oral PPIs for prophylaxis of drug-induced peptic ulcers focusing on the following three points: (1) prescription patterns of anti-ulcer agents, (2) clinical outcomes, i.e., the effectiveness for prophylaxis of drug-induced peptic ulcers, and (3) drug costs in the targeted population.

## Methods and materials

### Overview of the pharmacy formulary system on oral PPIs in KCH

Considering the efficacy, safety, economy, and volume of past prescriptions in our facility, we established a formulary system on oral PPIs, where generic lansoprazole (LPZ) was recommended as a first-choice PPI except for situations described below where using a stronger acid-suppressive agent, i.e. vonoprazan (VPZ), was recommended according to the Japanese clinical practice guidelines [12, 13]; (1) treatment for severe gastroesophageal reflux disease [13](2) prevention for peptic ulcer and/or bleeding in patients receiving dual antiplatelet therapy (DAPT), co-administration of two or more of anti-thrombotic agents, or administration of one anti-thrombotic agent with non-steroidal anti-inflammatory drugs (NSAIDs), and (3) eradication therapy for *Helicobacter pylori*. A physician (occasionally with patients) finally decided on the selection of PPIs without being forced by the formulary system. As a concrete operation, the policy was disseminated through notification and alerting a physician to the recommended information through a pop-up caution system in computerized prescriber order entry. If the first recommended PPI, LPZ, is selected, a pop-up alert system is not activated. When the other PPIs are selected, the alert will be displayed in both inpatient and outpatient settings. This formulary system was implemented in October 2020.

### Setting, population, and the study design

We conducted a retrospective cohort study. Although the formulary was applied to both therapeutic and prophylactic use of PPIs, we focused only on prophylactic use for drug-induced peptic ulcers in this study to evaluate the change in clinical outcomes of PPI use. In-hospital patients were targeted to evaluate the impact of cost changes considering the Japanese insurance system, i.e., Diagnosis Procedure Combination, a comprehensive payment system without charging each drug cost [14]. We reviewed the medical records of patients aged ≥ 18 years admitted to KCH from April 2020 to March 2021, corresponding to approximately six months before and after the implementation date of the formulary system on October 1, 2020. Prophylactic use was identified if anti-ulcer agents were administered within three days after prescription of any low-dose aspirin (LDA), anti-platelets (APL), or NSAIDs; these drugs are indicated for prophylactic anti-ulcer agent use in the Japanese guideline [12]. To detect new users of objected medications and at-risk populations for drug-induced peptic ulcers, exclusion criteria were as follows: (1) diagnosis with the International Classification of Disease 10th revision (ICD-10) codes K25x; gastric ulcer, K26x; duodenal ulcer, K27x; peptic ulcer, site unspecified, K28x; gastrojejunal ulcer, or K922; gastrointestinal haemorrhage at the admission, (2) prior use of histamine-2 receptor blocker, PPI, LDA, APL, or NSAIDs within 90 days before the admission, (3) discharge within 48 h of admission, (4) anti-ulcer agents for treatment within seven days from admission, and (5) no use of any of LDA, APL, or NSAIDs within seven days from admission. The study entry date of each patient was defined as the start date of any LDA, APL, or NSAIDs. The study design diagram is shown in Supplementary Figure S1.

The following information was collected; sex, age, body weight, height, diagnoses coded in ICD-10 codes, medications coded by the Japanese Ministry of Health, Labour and Welfare, records of treatment and procedures, and dates of admission and discharge. We used the first hospitalization data if a patient was hospitalized ≥ 2 times during the observation period.

### Exposure and outcomes

**Fig. 1:**
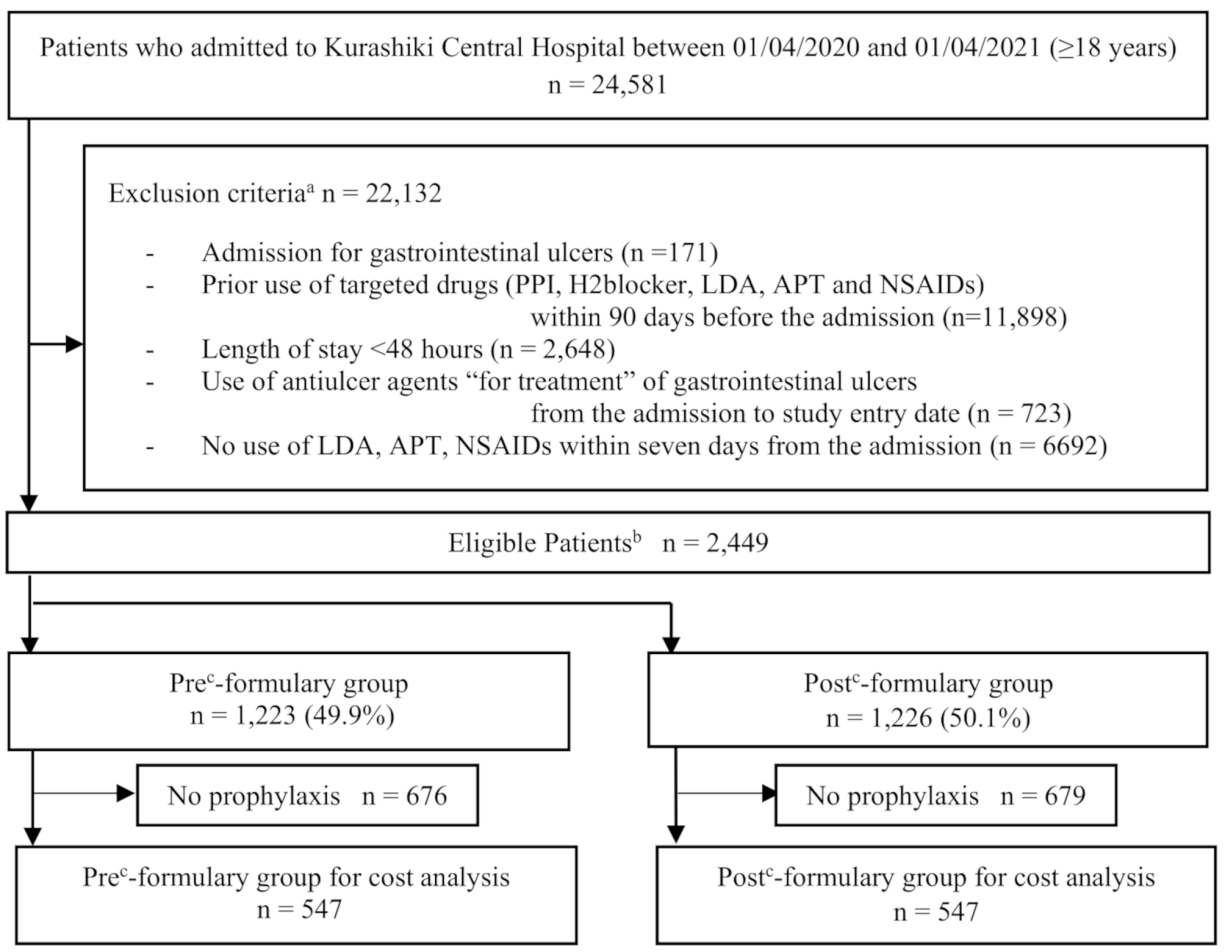
Flowchart of the present study. (**a**) Although we defined admission for *Helicobacter pylori* infection as an exclusion criterion in advance, no patient was observed. (**b**) In-patients who received low-dose aspirin, anti-platelets, or non-steroidal anti-inflammatory drugs within seven days from hospital admission. (**c**) The formulary system for PPI was applied officially on 10/01/2020 in the Kurashiki Central Hospital Abbreviations; PPI, proton pump inhibitor; LDA, low-dose aspirin; APT, anti-platelet therapy; NSAIDs, non-steroidal anti-inflammatory drugs

Exposure was the implementation of the PPI formulary system. Primary outcomes were changes in the following three before and after the exposure: (1) proportion of the first selected prophylactic agent within three days from the study entry date. This period was defined to identify the prophylactic use of the anti-ulcer agents. The proportion was calculated with the number of patients receiving any anti-ulcer agents as the numerator and the denominator as the number of eligible patients in each segment of 14 days, as described below in the statistical analysis. (2) Occurrence of drug-induced peptic ulcer within 30 days from the study entry date. The cases of drug-induced peptic ulcer were identified based on the ICD-10 diagnostic codes: K25x, K26x, K27x, K28x, and K922. We used the first event only if ≥ 2 events were observed during the same hospitalization in each patient. (3) The drug costs. The first and second outcomes were analyzed for all eligible patients classified into pre- and post-formulary groups, and the third outcome, the drug costs, was analyzed in the patients who received the prophylactic anti-ulcer agents (Fig. 1).

### Statistical analysis

We used an interrupted time series (ITS) analysis with a linear regression model for analyzing the prescription pattern of anti-ulcer agents before and after the exposure [15]. Considering the required time-point for the statistical model and the available data sample size [16, 17], we attempted to apply an autoregressive conditional heteroscedasticity, ARCH, model. Each time point was defined by dividing the study period into 14-day segments to ensure enough number of patients in each.

To investigate the effect on the clinical events, i.e., the occurrence of drug-induced peptic ulcer, we first compared the following items before and after exposure: demographic factors, body mass index (BMI), main diagnosis, Charlson comorbidity index (CCI) [18], and prophylactic prescription of anti-ulcer agents for drug-induced peptic ulcers; the differences between the groups were assessed with standardized differences. Although we considered differences less than 10% as negligible imbalances in baseline characteristics between the groups [19] and tried to apply propensity score analysis as required in advance, no differences were consequently observed. Because of the balanced characteristics of targeted groups, we determined not to adjust confounding factors in the main analysis, considering a conceptual model shown in Supplementary Figure S2. Thus, we evaluated the risk difference and the risk ratio with 95% confidence intervals (CI) by univariable analysis to estimate the association between the implementation of the formulary system and clinical events.

To evaluate changes in drug costs, we multiplied the medication days and daily medication costs based on the Japanese standard drug price (Supplementary Table S1). After confirming the distributions of medication days of anti-ulcer agents up to 90 days were comparable in each group, we compared the drug costs using the Mann-Whitney U test.

All statistical tests were two-tailed with a significance alpha level of 0.05 and all analyses were performed using SAS OnDemand for Academics (SAS Institute Inc., Cary, NC, USA).

### Sensitivity analysis

Four sensitivity analyses were performed to confirm the consistency of the main results. First, to consider potential biases due to outliers in ITS, an analysis excluding outliers identified in statistical processes was examined. Second, for the association between the intervention of the formulary and clinical outcome, we conducted an analysis defining the outcome as the diagnosis information plus the implementation of gastrointestinal endoscopy according to several previous studies focusing on “endoscopically confirmed” peptic ulcers [20–22]. Third, we used 60 or 90 days of the observation period to consider the effect of information bias. Finally, post-hoc, a multiple imputation method that derived 20 datasets was used [23], because we observed differences in the proportion of the missing data on BMI reportedly associated with upper gastrointestinal symptoms by sex [24]. Multivariable analyses were performed adding sex and BMI to the explanatory variable to evaluate potential biases due to the missing data. A modified least-squares regression for a risk difference [25] and a modified Poisson regression for a risk ratio [26] were used.

### Ad hoc analysis

Two ad hoc analyses were conducted to investigate the concrete part affected by the implementation of the formulary system. The first ad hoc analysis described the distribution of the selected prophylactic drugs by clinical departments. The other analysis examined the influence on drug-switching, in cases of change from the first chosen prophylactic drug to the second.

## Results

Figure 1 shows the flow of the present study. After applying the exclusion criteria to 24,581 in-patients, 2,449 who started LDA, APL, or NSAIDs within seven days of admission were eligible. Of these, 1,223 (49.9%) and 1,226 (50.1%) patients were included in the pre- and post-formulary groups, respectively. Table 1 shows the patients’ characteristics. There was no obvious deviation in the timing of patient admission (Supplementary Figure S3). The median age in the pre- and post-formulary group was 60 and 58 years, respectively, and males were 46.4% and 47.6%, respectively. In common with the groups, the use of NSAIDs was the most frequent drug for inclusion, followed by DAPT. At admission, malignant neoplasms and cardiovascular diseases were equally the most frequent. Although BMI data were missing in 821 (33.5%) of all the subjects, standardized differences in all characteristics were less than 10%. Therefore, the propensity score analysis was not performed.

**Table 1:** Patients’ characteristics at the study entry date

*N*, (%)		Pre-Formulary^a^ Total no. = 1,223		Post-Formulary^a^ Total no. = 1,226		Standardized Difference
Male		567	(46.4)	584	(47.6)	0.026
Age (median, IQR)		60	(41, 72)	58	(39, 72)	0.045
BMI (median, IQR)		23.3	(21.0, 26.1)	23.3	(20.8, 25.8)	0.005
Missing data of BMI		343	(28.1)	478	(39.0)	NR
Charlson Comorbidity Index (median, IQR)		1	(0, 2)	1	(0, 2)	0.035
Drugs for inclusion						
LDA		92	(7.5)	83	(6.8)	0.029
Antiplatelets		8	(0.7)	13	(1.1)	0.044
DAPT		123	(10.1)	112	(9.1)	0.034
NSAIDs		928	(75.9)	948	(77.3)	0.031
Combination of two categories^b^		47	(3.8)	42	(3.4)	0.022
Combination of three categories		25	(2.0)	28	(2.3)	0.016
Concomitant medications						
Gastric mucosal protective agents^c^		9	(0.7)	9	(0.7)	< 0.001
Anticoagulants		50	(4.1)	46	(3.8)	0.017
Systemic corticosteroids, oral		16	(1.3)	6	(0.5)	0.087
Systemic corticosteroids, injection		21	(1.7)	23	(1.9)	0.012
Bisphosphonates, oral		4	(0.3)	10	(0.8)	0.065
Bisphosphonates, injection		0	(0.0)	1	(0.1)	0.040
Antineoplastic agents, oral		9	(0.7)	11	(0.9)	0.018
Antineoplastic agents, injection		18	(1.5)	15	(1.2)	0.022
SSRI		2	(0.2)	5	(0.4)	0.046
Main disease categories in the hospitalizations						
Certain infectious and parasitic diseases		4	(0.3)	9	(0.7)	0.056
Neoplasms		308	(25.2)	297	(24.2)	0.022
Diseases of the blood and blood-forming organs and certain disorders involving the immune mechanism		1	(0.1)	1	(0.1)	< 0.001
Endocrine, nutritional and metabolic diseases		12	(1.0)	15	(1.2)	0.023
Mental and behavioural disorders		0	(0.0)	1	(0.1)	0.040
Diseases of the nervous system		6	(0.5)	13	(1.1)	0.065
Diseases of the eye and adnexa		9	(0.7)	4	(0.3)	0.056
Diseases of the ear and mastoid process		14	(1.1)	8	(0.7)	0.052
Diseases of the circulatory system		301	(24.6)	300	(24.5)	0.003
Diseases of the respiratory system		62	(5.1)	83	(6.8)	0.072
Diseases of the digestive system		123	(10.1)	116	(9.5)	0.020
Diseases of the skin and subcutaneous tissue		9	(0.7)	7	(0.6)	0.020
Diseases of the musculoskeletal system and connective tissue		60	(4.9)	67	(5.5)	0.025
Diseases of the genitourinary system		42	(3.4)	31	(2.5)	0.053
Pregnancy, childbirth and the puerperium		150	(12.3)	149	(12.2)	0.003
Congenital malformations, deformations and chromosomal abnormalities		8	(0.7)	4	(0.3)	0.047
Symptoms, signs and abnormal clinical and laboratory findings, not elsewhere classified		1	(0.1)	4	(0.3)	0.054
Injury, poisoning and certain other consequences of external causes		111	(9.1)	115	(9.4)	0.011
Coronavirus infectious disease 19		2	(0.2)	2	(0.2)	< 0.001

Abbreviations: PPI, Proton Pump Inhibitor; IQR, interquartile range; BMI, body mass index; LDA, low-dose aspirin; DAPT, dual antiplatelet therapy; NSAIDs, non-steroidal anti-inflammatory drugs; SSRI, selective serotonin reuptake inhibitor; ICD, international classification of disease; NR, not reported

^a^. The formulary system for PPI was applied officially on October 1, 2020 in the Kurashiki Central Hospital

^b^. Except for dual anti-platelet therapy

^c^. Drugs categorized as “A02BX” in World Health Organization Anatomical Therapeutic Codes

### ITS analysis of the prescription pattern of anti-ulcer agents

**Fig. 2:**
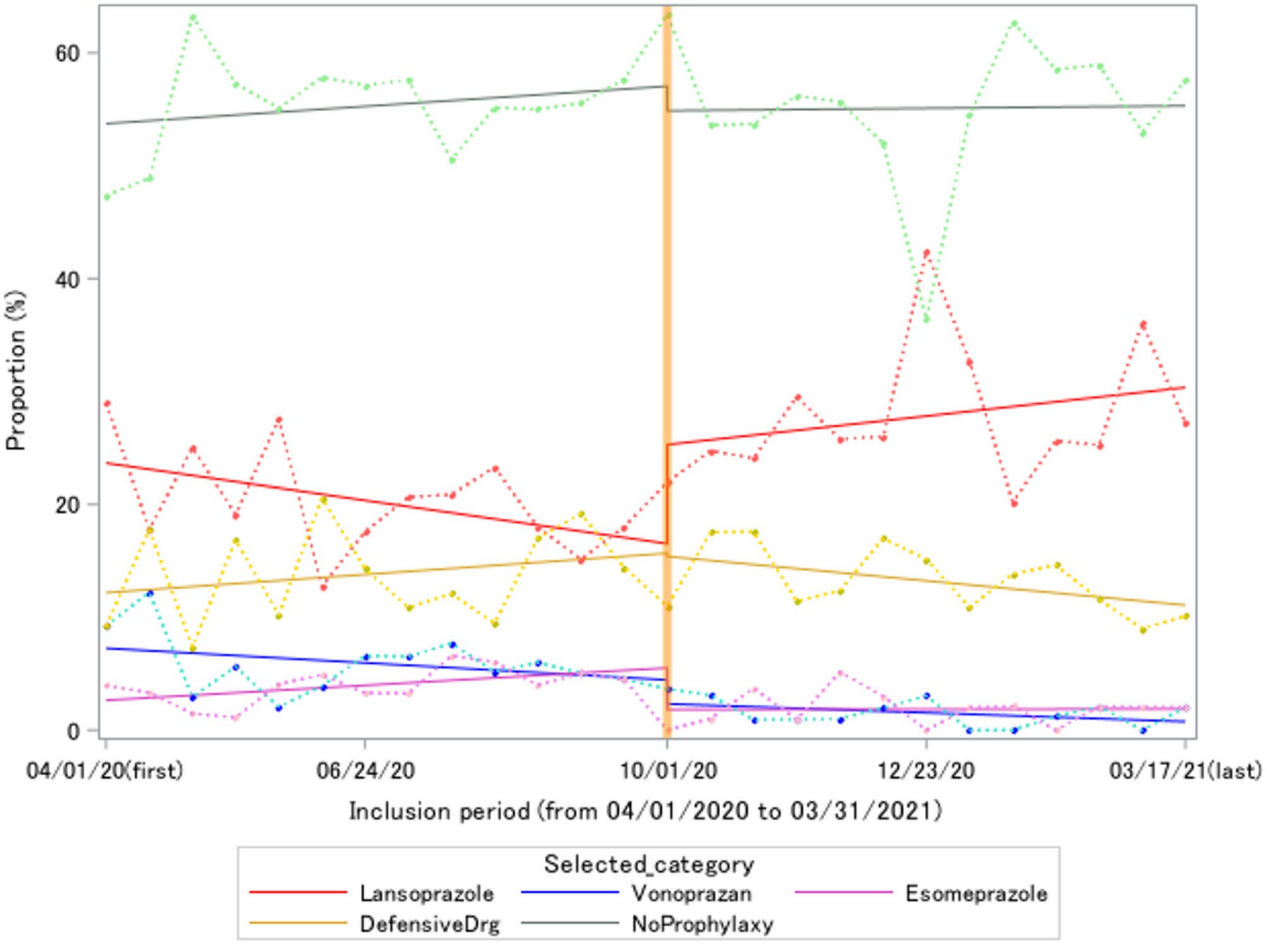
ITS analysis for the intervention of the PPI formulary system. The date in the X-axis represents the start date of the segment. The proportion of the selected categories in each segment was plotted with each broken line. Continuous lines represent an estimate from an ordinary least-square regression. The thick orange vertical line on October 1, 2020, indicates the point at which the formulary intervention began. The plots of rabeprazole and histamine 2 receptor antagonists were excluded in this figure due to low frequency

In the analysis of the prescription pattern of anti-ulcer agents, approximately 3–4% of the total patients were observed in each segment of 14 days (Supplementary Table S2). While no prophylactic medication was prescribed in 55.3% of the subjects, LPZ was the most selected anti-ulcer agent followed by some gastric mucosal protective agents, such as rebamipide or sucralfate. Trends in the proportion of first selected agents (including the no-use group) were plotted using regression lines, as shown in Fig. 2. When confirming the statistical parameters for time series data in the overall period, an obvious autocorrelation of the error term (Durbin-Watson test, *p* > 0.05) and changes in variance across time by using lag windows were not detected (The Q statistics test, *p* > 0.05). Given the white noise and normality of the error term, we did not apply an autoregression model but an ordinary linear square regression for these time series data. The level and trend changes in each drug between pre- and post-formulary intervention, estimated from the ordinary linear square, are shown in Table 2. An 8.75% (95% CI; 0.12, 17.38) increase in the level change of LPZ, a main targeted agent in the formulary, was observed. In the sensitivity analysis for ITS excluding the outlier, corresponding to the term ‘New Year holidays’, the results of level and trend change were consistent with the main ITS analysis (Supplementary Table S3 and Figure S4).

**Table 2:** Changes in the prophylaxis proportion of peptic ulcer from interrupted time series analysis

	Lansoprazole		Vonoprazane fumarate		Esomeprazole		Rabeprazole		Histamine receptor 2 blockers		Gastroprotective agents		Non-prophylaxis	
Intercept (β0)	24.19^a^	(17.74, 30.64)	7.47 ^a^	(4.99, 9.95)	2.42 ^a^	(0.64, 4.21)	0.53	(-0.02, 1.08)	-0.01	(-0.78, 0.76)	11.92 ^a^	(7.57, 16.26)	53.48^a^	(46.48, 60.48)
Baseline trend(β1)	-0.55	(-1.36, 0.26)	-0.22	(-0.53, 0.10)	0.22	(-0.004, 0.45)	-0.04	(-0.11, 0.03)	0.06	(-0.04, 0.16)	0.27	(-0.28, 0.82)	0.25	(-0.63, 1.14)
Level change (β2)	8.75^b^	(0.12, 17.38)	-2.12	(-5.45, 1.20)	-3.68^b^	(-6.08, -1.29)	0.15	(-0.58, 0.89)	-0.65	(-1.68, 0.38)	-0.28	(-6.09, 5.54)	-2.17	(-11.55, 7.20)
Slope change pre- to post-^c^ (β3)	0.97	(-0.18, 2.12)	0.08	(-0.36, 0.53)	-0.22	(-0.54, 0.10)	0.04	(-0.06, 0.14)	-0.03	(-0.17, 0.11)	-0.63	(-1.40, 0.15)	-0.22	(-1.47, 1.03)

^a^*P* < 0.05, an approximation to the significance probability for the intercept being zero

^b^*P* < 0.05, an approximation to the significance probability for the level change being zero

^c^ The formulary system of PPI was applied officially on October 1, 2020 in the Kurashiki Central Hospital

### Risk of drug-induced peptic ulcer

The results for the risk for drug-induced peptic ulcers are shown in Table 3. The defined 30-day events occurred in 21 (1.72%) patients in the pre-formulary group and 16 (1.31%) in the post-formulary group. Univariate analysis considering the causal model mentioned above confirmed a risk difference of -0.41% (95% CI: -1.38%, 0.55%) and a risk ratio of 0.996 (95% CI: 0.986, 1.006). The sensitivity analyses for outcome definition with the diagnosis information plus the implementation of esophagogastroduodenoscopy and for observation periods showed consistent results, with no increases in the events. The other sensitivity analysis with a multiple imputation method for missing data was consistent with the main result (risk difference, -0.38%; 95% CI [-1.34, 0.59] and risk ratio, 0.754; 95% CI [0.394, 1.445]).

**Table 3:** The association between the intervention of PPI formulary and the incidence of drug-induced peptic ulcer

		Pre-formulary			Post-formulary			Risk difference (%) (95% CI)	Risk ratio (95% CI)
		No. of events^a^	(%)		No. of events^a^	(%)			
Main analysis^b^		21	(1.72)		16	(1.31)		-0.41 (-1.38, 0.55)	0.996 (0.986, 1.006)
Sensitivity analysis									
	Outcome definition^c^	2	(0.16)		2	(0.16)		0.00 (-0.32, 0.32)	1.000 (0.997, 1.003)
	Observation period^d^ 60 days	23	(1.88)		25	(2.04)		0.16 (-0.94, 1.26)	1.002 (0.991, 1.013)
	Observation period^d^ 90 days	28	(2.29)		27	(2.20)		0.09 (-1.26, 1.09)	0.999 (0.987, 1.011)
	Multiple imputation	21	(1.72)		16	(1.31)		-0.38 (-1.34, 0.59)^e^	0.754 (0.394, 1.445)^f^

Abbreviations: PPI, proton pump inhibitor

^a^ If ≥ 2 events were observed during the same hospitalization in each patient, only the first event was considered

^b^ Univariable analyses were conducted considering a directed acyclic graph shown in Supplementary Figure S2

^c^ The outcome was defined as the diagnosis information plus the implementation of esophagogastroduodenoscopy because the validity of peptic ulcer identification using Japanese administrative data was absent

^d^ Sixty or 90 days from the study entry date was the observation period to consider an effect of information bias

^e^ A modified least-squares regression was conducted. An intervention of the formulary system and BMI was applied as an explanatory variable, considering a directed acyclic graph shown in Supplementary Figure S2

^f^ A modified Poisson regression was conducted. An intervention of the formulary system and BMI was applied as an explanatory variable, considering a directed acyclic graph shown in Supplementary Figure S2

### Evaluation of cost-changes

The number of patients who received the prophylactic agents for drug-induced peptic ulcer was 1,094 (44.7%) in total. Distribution of the medication days was similar between the pre- and post-formulary groups in terms of cost analysis (Supplementary Figure S5, *P*  = 0.62). Consequently, 156,055.2 JPY per 90 days, corresponding to about $1,000 per 90 days at an exchange rate of 150 JPY to $1, was saved as drug costs ( *p*  < 0.01) (Supplementary Table S4 ). When calculating an incremental cost-effectiveness ratio (ICER) per 90-day measurement period based on the estimates of 0.09% of risk difference from a sensitivity analysis (i.e., a negative value of effects) as the denominator, and a saving cost of $1,040.4 as the numerator, an ICER = $11,560 per event reduction per 100 patients.

### Ad hoc analysis

An ad hoc analysis investigating an influence on prescription patterns by clinical departments found that an impact of formulary policy may have contributed more to surgical departments (Supplementary Table S5), while initial prophylactic drugs were rarely switched and no clear trends by the policy were observed in the other analysis (Supplementary Table S6).

## Discussion

The present study showed that the intervention of the formulary system on prophylactic use of oral PPIs for drug-induced peptic ulcers contributed to the aimed change in level and trend of prescription patterns, without increasing the event risk, and with a slight positive cost saving.

In contrast to previous studies examining the change by formulary interventions [9–11], we performed an ITS analysis to evaluate the aimed change in “level” and “trend” by the intervention with the clear timing of the implementation. The proportion of the first recommended PPI, LPZ, was the most positive change among available PPIs throughout the observation period. Before formulary implementation, a moderately decreasing trend in LPZ selection was observed, but it turned positive in the post-intervention period with a significant level change. Meanwhile, a high-cost comparator, VPZ, showed no statistically significant change. This may have been affected by the small baseline proportion of VPZ selection before the formulary intervention. Generally, the ITS analysis to estimate the longitudinal effectiveness of an intervention requires some assumptions; linear trends in pre-intervention, invariance of population characteristics over the observation period, and no effects other than the intervention [27]. Our formulary system and its analysis likely met them because we could visualize linearity without heteroscedasticity and observed invariance of population characteristics by descriptive statistics, and no other policy on the use of PPIs (such as a new clinical practice guideline disclosure) was implemented during the study period in the facility. In the regression line after the formulary implementation, a rapid increase in LPZ selection proportion and a decrease in no prophylaxis were observed during the New Year’s holiday. In this period, the practices were likely influenced by changes in the staffing or temporary discharge of inpatients, assessed as outliers by statistical analyses. Considering consistent results from the sensitivity analysis for outlier data, ITS analyses revealed that the aim of the formulary system to guide the prescription pattern was achieved.

While the purpose of changing the prescription patterns was accomplished, the actual prophylactic administration of anti-ulcer agents was less than 50% of all subjects. One of the reasons may be that physicians considered the PPI-associated adverse events more than a prophylactic benefit [28]. Alternatively, many patients taking NSAIDs for a short term of less than 8 days were included (about 75% of total subjects, Supplementary Figure S6); the duration of medication was one of the risk factors of NSAID-induced ulcers in a meta-analysis [29]. Due to the expected short-term of medication, a prophylaxis might not be implemented. In this study, we included patients with more than one day of NSAID use, because the situation was a potential risk for drug-induced peptic ulcers [30].

The number of drug-induced peptic ulcers was about 1–2% in both pre- and post-term, without a statistically significant risk difference and a risk ratio. The actual proportion of the events in this study was lower than in some previous studies reporting a prevalence of LDA-induced peptic ulcer [20, 22]. Since the prevalence of peptic ulcer has decreased over the years in Japan [31], differences in the timing of each study from this study may have affected the results. However, sensitivity analyses focusing on endoscopically confirmed peptic ulcers or applying different observation periods confirmed the consistency of the results, while the proportion of new occurrences decreased over time. Approximately 30% of data were missing for patients’ BMI in the present study. A multicenter cohort study reported the relationship between BMI and upper gastric symptoms by sex [24]. We were concerned that if the distribution of missing data was unbalanced in each population, it could affect the results (Supplementary Figure S2). The multivariable sensitivity analysis using multiple imputations showed consistent results with the main analysis. Taken together, we concluded that the formulary intervention did not increase the risk of drug-induced peptic ulcer.

 We confirmed a positive drug cost saving by the formulary intervention; however, its impact was slight. Some studies focusing on cost change reported the effectiveness of the formulary on PPIs [ 9, 32– 34 ]. However, since the aims and settings are different in each scene, comparing the figures of cost evaluation among these studies may be inappropriate. Cost savings for drugs in this study were about $1,000 per 90 days, calculated at $330 per month with an exchange rate of 150 JPY to $1. A total of 547 patients with prophylactic use of anti-ulcer agents in each group were analyzed for cost change; the impact of the individual cost-saving was $0.6 (95.1 JPY) per patient per month. Moreover, a value of ICER itself may be uncertain, since the point estimates of the denominator, the risk differences, were consistently small and near zero, and its confidence interval crossed zero [ 35 ]. However, unlike the U.S. and other countries, the Japanese ministry provides national health insurance coverage. When assuming that the event risk difference within 5% was clinically acceptable, the risk was equivalent before and after the formulary intervention, according to the confidence intervals from our analyses. Therefore, even such a slight cost-saving through the change of the prescription pattern is meaningful from the perspective of national economic policy.

The present study has some limitations that warrant further consideration. First, there was the limitation of the condition in ITS modeling. Several conditions such as the number of time points and average sample size per time point are required in ITS [36]. Although we configured the observation period corresponding to 12 time points in the pre- and post-intervention period by dividing six months in each period by every two weeks, the statistical power might be inadequate according to these conditions. We considered extending the observation period, however, the supply shortage of LPZ occurred since the middle of 2021 in Japan [37]. Thus, data from this period might not have met the assumptions required in ITS [27]. For the same reason, the analyses could not adjust for a potential seasonality, although no autocorrelation throughout the observation period was evaluated. However, this was unlikely because an intention for PPI selection seems not to depend on seasonality. Second, as for clinical event risk evaluation, the sample size might not be large enough to detect a small occurrence of peptic ulcers. Given the absence of facilities with a shared formulary system, we could not include more samples in this study. Third, for cost evaluation, we could not assess the overall medical costs of individual cases and the cost-benefit balance considering the quality-adjusted life year. Finally, the results of the present study may have limited generalizability. The impact of the formulary system may depend on settings where the insurance system differs from that in Japan. In addition, because this was single-center research, the results may have depended on the communication between the pharmacy and clinical departments. While an ad hoc analysis showed a possible effect of the formulary policy on surgical departments in this study, the part most affected by a formulary system can be different at other facilities. However, similar effectiveness can be demonstrated at many hospitals since general pharmaceutical care guidelines are practiced in KCH and other hospitals across the country.

## Conclusions

The intervention of our formulary system on oral PPI use contributed to the aimed change of level and trend of prescription patterns without an apparent increase in the event risk among eligible patients for prophylactic anti-ulcer medication. Although small, drug costs showed slight savings.

## Electronic supplementary material

Below is the link to the electronic supplementary material.

Supplementary Material 1

## Data Availability

The datasets generated and analyzed during the current study are not publicly available due to the data-handling terms and conditions at the facility but may be available from the corresponding author on reasonable request.

## Abbreviations

APL: Antiplatelets
BMI: Body mass index
CCI: Charlson comorbidity index
CI: Confidence interval
DAPT: Dual anti-platetlet therapy
ICD: International classification of diagnosis
ITS: Interrupted time series
JPY: Japanese yen
KCH: Kurashiki central hospital
LDA: Low-dose aspirin
LPZ: Lansoprazole
NSAID: Non-steroidal anti-inflammatory drug
PPI: Proton pump inhibitor
VPZ: Vonoprazan
