# Supplementary material for: Effectiveness of a formulary system on prophylactic oral proton pump inhibitors for drug-induced peptic ulcer in a Japanese tertiary hospital: an interrupted time series analysis
Source: J Pharm Health Care Sci. 2025 Jun 18;11:51. doi: 10.1186/s40780-025-00459-w (PMC12175457; doi:10.1186/s40780-025-00459-w)
Supplement: Supplementary file 1 — Supplementary Material 1 [file 40780_2025_459_MOESM1_ESM.docx]

Supplementary Table S1. Formulary and price list of antiulcer agents in Kurashiki Central Hospital (as of 09/01/2021)

| ATC code^a^ | General name | Trade name | Formulation | Price (JPY) |
| --- | --- | --- | --- | --- |
| A02BC03 | Lansoprazole^b^ | Lansoprazole OD 15mg [SAWAI]^c^ | Tablets | 19.3 |
|  |  | Lansoprazole OD 30mg [SAWAI]^c^ | Tablets | 33.4 |
| A02BC08 | Vonoprazan Fumarate | Takecab 10mg | Tablets | 125.0 |
|  |  | Takecab 20mg | Tablets | 187.5 |
| ― | Aspirin + Vonoprazan Fumarate | Cabpirin combination | Tablets | 126.7 |
| A02BC05 | Esomeprazole Magnesium Hydrate | Nexium 10mg | Granules for Suspension | 71.8 |
|  |  | Nexium 10mg | Capsules | 62.7 |
|  |  | Nexium 20mg | Capsules | 108.9 |
| A02BC04 | Rabeprazole Sodium | Pariet 10mg | Tablets | 72.7 |
|  |  | Pariet 20mg | Tablets | 136.2 |
| A02BA03 | Famotidine | BlostarM tab.20 ^c^  (up to 2020/08/10) | Tablets | 10.1 |
|  |  | Famotidine D 20mg [NICHIIKO] ^c^  (from 2020/08/11) | Tablets | 10.1 |
|  |  | Gaster 10% | Powder | 155 (/g) |
| A02BA08 | Lafutidine | Protecadin 10mg | Tablets | 24.9 |
| A02BA04 | Nizatidine | Acinon 75mg | Tablets | 15.7 |
| A02BC03 | Lansoprazole | Takepron Intravenous | Injection | 383.0 |
| A02BC01 | Omeprazole Sodium | Omeprazole for Intravenous | Injection | 217.0 |

^a^ ATC codes defined by the World Health Organization

^b^ First recommended drug for prophylaxis of secondary peptic ulcer in the formulary system of Kurashiki Central Hospital

^c^ A generic agent

| Supplementary Table S2. The distribution of participants in each segment^a^ | | | | | | | | | | | | | | | | | | | | | | | | | | |
| --- | --- | --- | --- | --- | --- | --- | --- | --- | --- | --- | --- | --- | --- | --- | --- | --- | --- | --- | --- | --- | --- | --- | --- | --- | --- | --- |
| Seg^a^ | 1 | 2 | 3 | 4 | 5 | 6 | 7 | 8 | 9 | 10 | 11 | 12 | 13 | 14 | 15 | 16 | 17 | 18 | 19 | 20 | 21 | 22 | 23 | 24 | 25 | 26 |
| No. Pt | 76 | 90 | 68 | 89 | 98 | 102 | 91 | 92 | 91 | 116 | 100 | 99 | 111 | 82 | 97 | 108 | 105 | 97 | 100 | 66 | 101 | 94 | 82 | 95 | 100 | 99 |
| N,[%] | [3.1] | [3.7] | [2.8] | [3.6] | [4.0] | [4.2] | [3.7] | [3.8] | [3.7] | [4.7] | [4.1] | [4.0] | [4.5] | [3.4] | [4.0] | [4.4] | [4.3] | [4.0] | [4.1] | [2.7] | [4.1] | [3.8] | [3.4] | [3.9] | [4.1] | [4.0] |
| LPZ | 22 | 16 | 17 | 17 | 27 | 13 | 16 | 19 | 19 | 27 | 18 | 15 | 20 | 18 | 24 | 26 | 31 | 25 | 26 | 28 | 33 | 19 | 21 | 24 | 36 | 27 |
| N,(%) | (29.0) | (17.8) | (25.0) | (19.1) | (27.6) | (12.8) | (17.6) | (20.7) | (20.9) | (23.3) | (18.0) | (15.2) | (18.0) | (22.0) | (24.7) | (24.1) | (29.5) | (25.8) | (26.0) | (42.4) | (32.7) | (20.2) | (25.6) | (25.3) | (36.0) | (27.3) |
| VPZ | 7 | 11 | 2 | 5 | 2 | 4 | 6 | 6 | 7 | 6 | 6 | 5 | 5 | 3 | 3 | 1 | 1 | 1 | 2 | 2 | 0 | 0 | 1 | 2 | 0 | 2 |
| N,(%) | (9.2) | (12.2) | (2.9) | (5.6) | (2.0) | (3.9) | (6.6) | (6.5) | (7.7) | (5.2) | (6.0) | (5.0) | (4.5) | (3.7) | (3.1) | (0.9) | (1.0) | (1.0) | (2.0) | (3.0) | (0.0) | (0.0) | (1.2) | (2.1) | (0.0) | (2.0) |
| EPZ | 3 | 3 | 1 | 1 | 4 | 5 | 3 | 3 | 6 | 7 | 4 | 5 | 5 | 0 | 1 | 4 | 1 | 5 | 3 | 0 | 2 | 2 | 0 | 2 | 2 | 2 |
| N,(%) | (4.0) | (3.3) | (1.5) | (1.1) | (4.1) | (4.9) | (3.3) | (3.3) | (6.6) | (6.0) | (4.0) | (5.1) | (4.5) | (0.0) | (1.0) | (3.7) | (1.0) | (5.1) | (3.0) | (0.0) | (2.0) | (2.1) | (0.0) | (2.1) | (2.0) | (2.0) |
| RPZ | 1 | 0 | 0 | 0 | 1 | 0 | 0 | 0 | 0 | 1 | 0 | 0 | 0 | 0 | 0 | 0 | 0 | 0 | 0 | 1 | 0 | 0 | 0 | 0 | 0 | 0 |
| N,(%) | (1.3) | (0.0) | (0.0) | (0.0) | (1.0) | (0.0) | (0.0) | (0.0) | (0.0) | (0.9) | (0.0) | (0.0) | (0.0) | (0.0) | (0.0) | (0.0) | (0.0) | (0.0) | (0.0) | (1.5) | (0.0) | (0.0) | (0.0) | (0.0) | (0.0) | (0.0) |
| H2RA | 0 | 0 | 0 | 0 | 0 | 0 | 1 | 1 | 2 | 0 | 0 | 0 | 1 | 0 | 0 | 0 | 1 | 0 | 0 | 1 | 0 | 1 | 0 | 0 | 0 | 1 |
| N,(%) | (0.0) | (0.0) | (0.0) | (0.0) | (0.0) | (0.0) | (1.1) | (1.1) | (2.2) | (0.0) | (0.0) | (0.0) | (0.9) | (0.0) | (0.0) | (0.0) | (1.0) | (0.0) | (0.0) | (1.5) | (0.0) | (1.1) | (0.0) | (0.0) | (0.0) | (1.0) |
| Other^b^ | 7 | 16 | 5 | 15 | 10 | 21 | 13 | 10 | 11 | 11 | 17 | 19 | 16 | 9 | 17 | 19 | 12 | 12 | 17 | 10 | 11 | 13 | 12 | 11 | 9 | 10 |
| N,(%) | (9.2) | (17.8) | (7.4) | (16.9) | (10.2) | (20.6) | (14.3) | (10.9) | (12.1) | (9.5) | (17.0) | (19.2) | (14.4) | (11.0) | (17.5) | (17.6) | (11.4) | (12.4) | (17.0) | (15.2) | (10.9) | (13.8) | (14.6) | (11.6) | (9.0) | (10.1) |
| No Pr | 36 | 44 | 43 | 51 | 54 | 59 | 52 | 53 | 46 | 64 | 55 | 55 | 64 | 52 | 52 | 58 | 59 | 54 | 52 | 24 | 55 | 59 | 48 | 56 | 53 | 57 |
| N,(%) | (47.4) | (48.9) | (63.2) | (57.3) | (55.1) | (57.8) | (57.1) | (57.6) | (50.6) | (55.2) | (55.0) | (55.6) | (57.7) | (63.4) | (53.6) | (53.7) | (56.2) | (55.7) | (52.0) | (36.4) | (54.5) | (62.8) | (58.5) | (59.0) | (53.0) | (57.6) |

Abbreviations; Seg; segment, LPZ; Lansoprazple, VPZ; Vonoprazan fumatate, EPZ; Esomeprazol, RPZ; Rabeprazole, H2RA; histamine receptor 2 antagonists, No Pr; No prophylaxis

^a^ Each time point was defined by dividing the study period into 14-day segments

^b^ This category has a gastromucosal defensive agent, such as rebamipide, teprenone, misoprostol, polaprezinc, and sucralfate hydrate

Supplementary Table S3 (related to Supplementary Figure S4). Sensitivity analysis considering the outliers, focusing on only the lansoprazole and non-prophylaxis population

|  | Lansoprazole | | Non-prophylaxis | |
| --- | --- | --- | --- | --- |
| Intercept (β0) | 24.19^a^ | (18.96, 29.42) | 53.48^a^ | (48.50, 58.46) |
| Baseline trend (β1) | -0.55 | (-1.21, 0.11) | 0.25 | (-0.37, 0.88) |
| Level change (β2) | 7.53^b^ | (0.49, 14.57) | -0.61 | (-7.31, 6.09) |
| Slope change ^c^ (β3) | 0.97 | (0.04, 1.90) | -0.22 | (-1.11, 0.67) |

^a^ *P* < 0.05, an approximation to the significance probability for the intercept being zero

^b^ *P* < 0.05, an approximation to the significance probability for the level change being zero

^c^ The formulary system was applied officially on 10/01/2020 in the Kurashiki Central Hospital

Supplementary Table S4. Estimation of changes in drug costs. Patients in this analysis were confined to prophylactic drug users

| Japanese Yen | Pre formulary group  (N = 547)^a^ | Post formulary group  (N = 547)^a^ | Cost difference |
| --- | --- | --- | --- |
| Lansoprazole | 169363.0 | 208988.4 | 39625.4 |
| Vonoprazan fumarate | 221657.1 | 52904.5 | -168752.6 |
| Esomeprazole | 102228.7 | 68901.6 | -33327.1 |
| Rabeprazole | 4907.7 | 7749.0 | 2841.3 |
| Histamine receptor 2 blocker | 727.2 | 848.4 | 121.2 |
| Gastroprotective agents | 61323.2 | 64759.8 | 3436.6 |
| Total prophylaxis drug costs | 560206.9 | 404151.7 | -156055.2 |

^a^ The formulary system was applied officially on 10/01/2020 in the Kurashiki Central Hospital

Supplementary Table S5. Distribution of the first selection of prophylactic drugs by the clinical department

| n, % | Gastro-protective agents | LPZ | VPZ | EPZ | RPZ | H2RA | Total |
| --- | --- | --- | --- | --- | --- | --- | --- |
| Internal medicine | 15 (4.5) | 251 (43.0) | 23 (25.6) | 38 (51.4) | 4 (100.0) | 0 (0.0) | 331 (30.3) |
| Preformulary period^a^ | 8 [53.3] | 120 [47.8] | 13 [56.5] | 21 [55.3] | 3 [75.0] | 0 [0.0] | 165 [49.8] |
| Postformulary period^a^ | 7 [46.7] | 131 [52.0] | 10 [43.5] | 17 [44.7] | 1 [25.0] | 0 [0.0] | 166 [50.2] |
|  |  |  |  |  |  |  |  |
| Surgical group^b^ | 318 (95.5) | 333 (57.0) | 67 (74.4) | 36 (48.6) | 0 (0.0) | 9 (100.0) | 763 (69.7) |
| Preformulary period^a^ | 163 [51.3] | 126 [37.8] | 59 [88.1] | 29 [80.6] | 0 [0.0] | 5 [55.6] | 382 [50.1] |
| Postformulary period^a^ | 155 [48.7] | 207 [62.2] | 8 [11.9] | 7 [19.4] | 0 [0.0] | 4 [44.4] | 381 [49.9] |
|  |  |  |  |  |  |  |  |
| Total | 333 | 584 | 90 | 74 | 4 | 9 | 1094 |

Abbreviations; LPZ; Lansoprazole, VPZ; Vonoprazan fumatate, EPZ; Esomeprazol, RPZ; Rabeprazole, H2RA; histamine receptor 2 antagonists

^a^ The formulary system was applied officially on 10/01/2020 in the Kurashiki Central Hospital

^b^ The surgical group included the department of critical care medicine, dermatology, obstetrics and gynecology, ophthalmology, otolaryngology, urology, and each surgery.

Figures in parentheses indicate the proportion of selections in the department in each drug group and figures in square brackets indicate the proportion of selections before and after formulary intervention in each department group.

Supplementary Table S6. The prophylactic drug switching from the initial medication to the second^a^

| n, (%) | Pre formulary  (N = 43)^b^ | Post formulary  (N = 53)^b^ | *P* value |
| --- | --- | --- | --- |
| Switching between PPIs | 22 (51.2) | 26 (49.1) | 1.000 |
| From VPZ to LPZ | 7 (16.3) | 3 (5.7) | 0.106 |
| From VPZ to EPZ | 2 (4.7) | 0 (0.0) | 0.198 |
| From LPZ to VPZ | 4 (9.3) | 4 (7.5) | 1.000 |
| From LPZ to EPZ | 7 (16.3) | 12 (22.6) | 0.607 |
| From LPZ to RPZ | 0 (0.0) | 4 (7.5) | 0.125 |
| From EPZ to LPZ | 1 (2.3) | 3 (5.7) | 0.625 |
| From EPZ to VPZ | 1 (2.3) | 0 (0.0) | 0.448 |
| From PPIs to the other category^c^ | 12 (27.9) | 22 (41.5) | 0.201 |
| From gastroprotective drug to PPI | 9 (20.9) | 5 (9.4) | 0.149 |

Abbreviations; PPI; proton pump inhibitor, VPZ; Vonoprazan fumatate, LPZ; Lansoprazole, EPZ; Esomeprazol, RPZ; Rabeprazole,

^a^ No switchings were observed in the case of initial selection of rabeprazole and histamine receptor 2 antagonists.

^b^ The formulary system was applied officially on 10/01/2020 in the Kurashiki Central Hospital.

^c^ This category included gastroprotective drugs or histamine receptor 2 antagonists.

Supplementary Figure S1


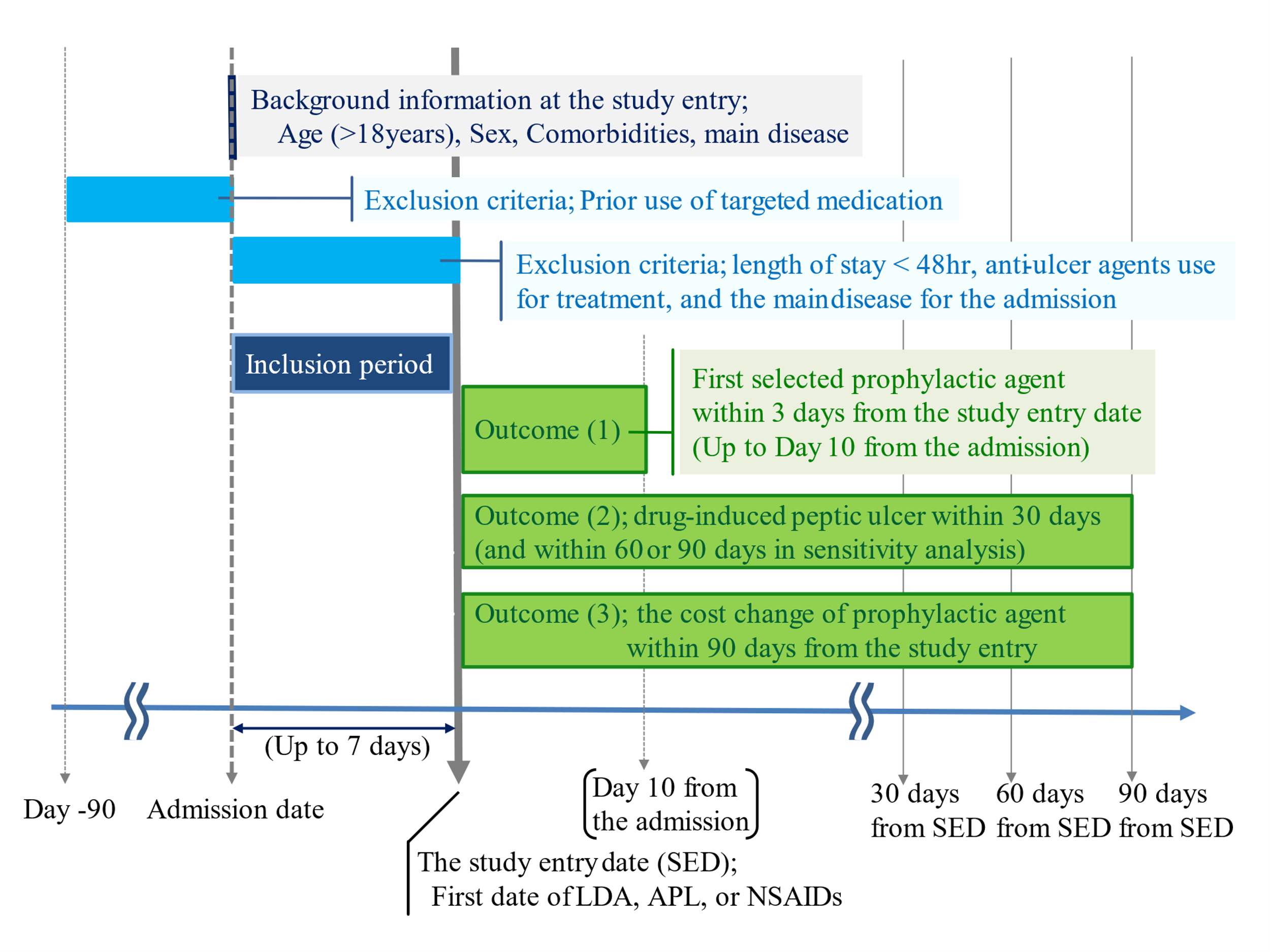


Supplementary Figure S1. Study design diagram

Supplementary Figure S2


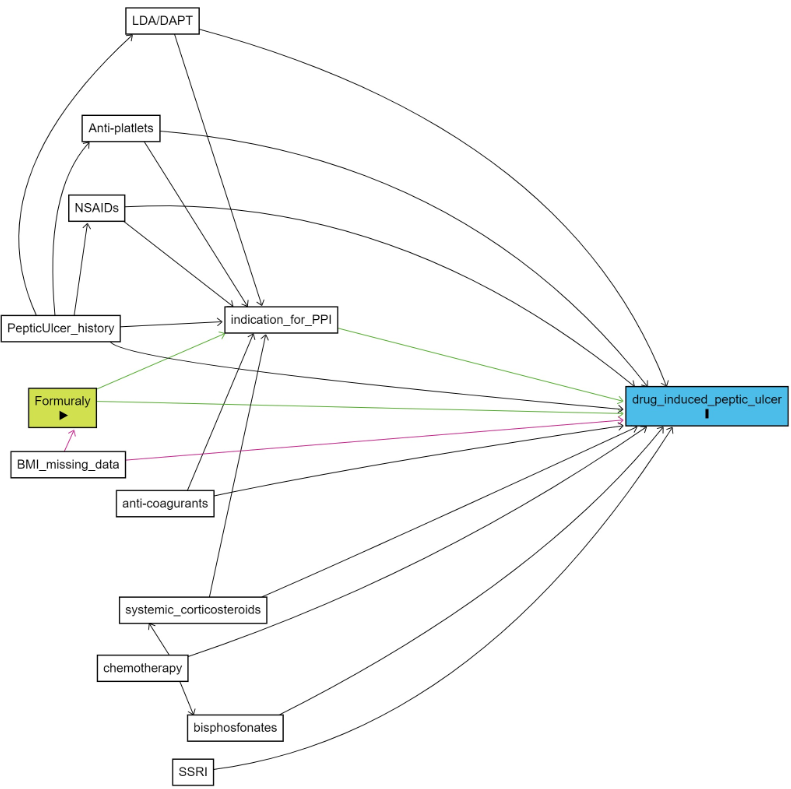


Supplementary Figure S2. A conceptual model represented by a directly acyclic graph (DAG) to evaluate an association between an intervention of the formulary system and a drug-induced peptic ulcer (drawn through Dagitty, <https://www.dagitty.net/>, see also Johannes Textor, Benito van der Zander, Mark K. Gilthorpe, Maciej Liskiewicz, George T.H. Ellison. Robust causal inference using directed acyclic graphs: the R package 'dagitty'. *International Journal of Epidemiology* 45(6):1887-1894, 2016.) Green arrows represent a direct causal path of interest, the yellow-green square (▶) represents the exposure, and the blue square (I) represents the outcome. The DAG that we assumed did not require adjustment or conditioning on any variables to be considered to estimate the causal relation between the exposure and the outcome, although an unbalanced proportion of the missing BMI associated with upper gastrointestinal symptoms can produce a biased path represented by red arrows.

Supplementary Figure S3


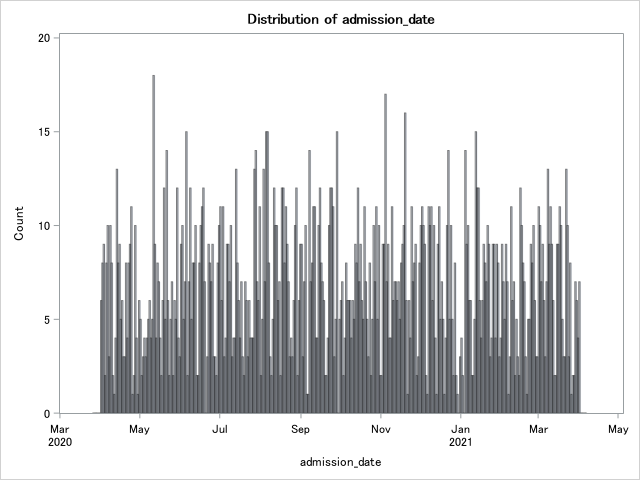


Supplementary Figure S3. Distribution of admission dates in each patient

Supplementary Figure S4


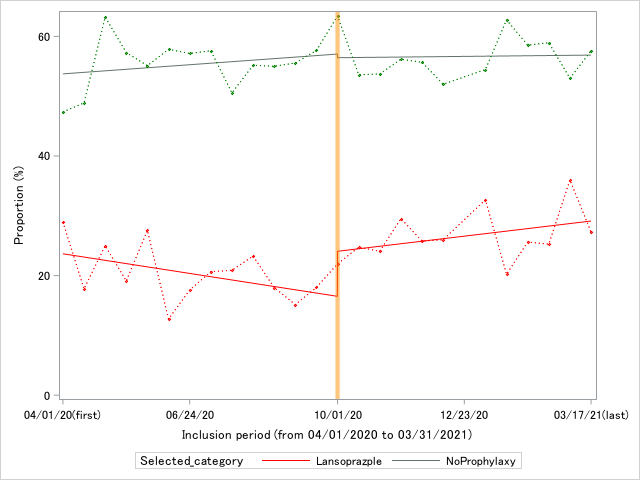


Supplementary Figure S4 (related to Supplementary Table 3). Sensitivity analysis considering the outliers, focusing on only the lansoprazole and non-prophylaxis population. The date on the X-axis represents the start date of each segment. Segment 20 starting on 12/23/20, which corresponded to the term of New Year holidays, was excluded from this sensitivity analysis. The proportion of the selected categories in each segment was plotted with each broken line. Continuous lines represent an estimate of the value of the proportion from an ordinary least-square regression. The thick orange vertical line on October 1, 2020, indicates the point at which the formulary intervention began.

Supplementary Figure S5


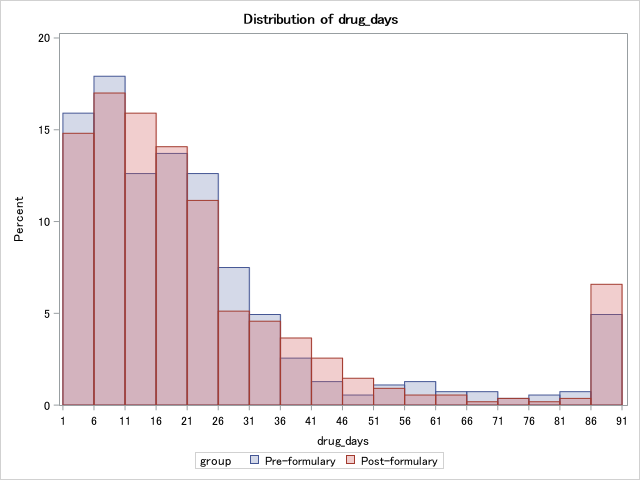


Supplementary Figure S5. The distribution of prophylactic agent days confining to the applied population. The median days (with interquartile range) in pre- and post-formulary group were 16 days (7, 27) and 16 days (8, 28), respectively. (Mann-Whitney U test, *P* = 0.62)

Supplementary Figure S6


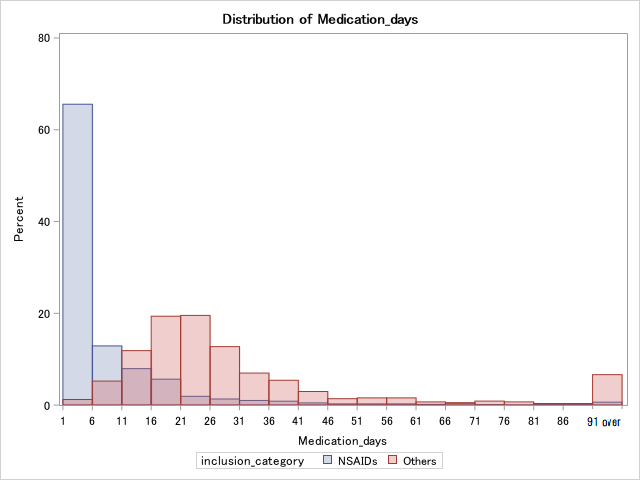


Supplementary Figure S6. The distribution of days of medication for inclusion criteria, which were low-dose aspirin, anti-platelets, or non-steroidal anti-inflammatory drugs.

The median days (with interquartile range) for NSAIDs and other medications were 3 (1, 8) and 23 days (17, 34), respectively.
